# Gnotobiotic Rodents: An *In Vivo* Model for the Study of Microbe–Microbe Interactions

**DOI:** 10.3389/fmicb.2016.00409

**Published:** 2016-03-31

**Authors:** Rebeca Martín, Luis G. Bermúdez-Humarán, Philippe Langella

**Affiliations:** Micalis Institute, INRA, AgroParisTech, Université Paris-SaclayJouy-en-Josas, France

**Keywords:** germ-free, gnotobiology, bacterial interactions, intestinal bacteria, microbiota

## Abstract

Germ-free rodents have no microorganisms living in or on them, allowing researchers to specifically control an animal’s microbiota through the direct inoculation of bacteria of interest. This strategy has been widely used to decipher host–microbe interactions as well as the role of microorganisms in both (i) the development and function of the gut barrier (mainly the intestinal epithelium) and (ii) homeostasis and its effects on human health and disease. However, this *in vivo* model also offers a more realistic environment than an assay tube in which to study microbe–microbe interactions, without most of the confounding interactions present in the intestinal microbiota of conventionally raised mice. This review highlights the usefulness of controlled-microbiota mice in studying microbe–microbe interactions. To this end, we summarize current knowledge on germ-free animals as an experimental model for the study of the ecology and metabolism of intestinal bacteria as well as of microbe–microbe interactions.

## Introduction

Like human beings, conventional rodents harbor trillions of bacteria and viruses (Williams, 2014). Germ-free (GF) animals, instead, are completely free of these microbes. In the field of gnotobiology [from the Greek “*gnōtos”* (known) and *“biotic”* (life)] (Gustafsson, 1959), such animals are used to study the effects of inoculation with specific, known microbes. To raise GF mice, researchers must separate pups from their mother’s wombs surgically, by aseptic cesarean or hysterectomy, thus avoiding contact with microorganisms present in the mother’s vagina and skin. Alternatively, GF pups can also be generated via the process of embryo transfer, which occurs in an isolator environment and thus enables the implantation of cleansed embryos into GF recipients under well-controlled conditions. A recipient female gives birth normally and cares for the offspring as if they were her own pups, thus enhancing the pups’ survival rate. GF pups are kept in a sterile environment their whole lives. In order to guarantee GF status, recurrent contamination tests are performed on their feces. Once GF animals have been produced, it is possible to expand the colony by crossing GF individuals. The new members of the colony have a natural birth and continue their life under the same sterile conditions as their progenitors.

**FIGURE 1:**
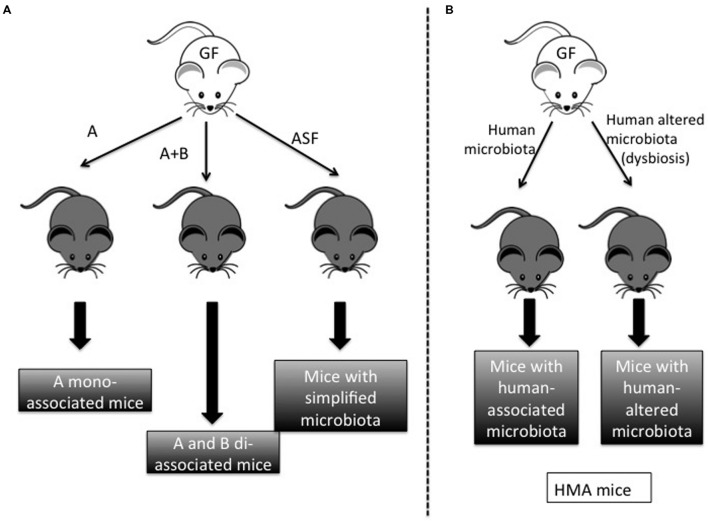
**Different models derived from germ-free (GF) mice. (A)** Gnotobiotic models: GF mice can be colonized by one bacterium (microorganism A) in order to obtain A-mono-associated mice. Similarly, GF mice can be colonized by microorganisms A and B to obtain A + B di-associated mice. When mice are colonized by a basic microbiota such as the altered Schaedler Flora (ASF), we obtain mice with a simplified microbiota. **(B)** Non-gnotobiotic models: when mice are colonized by human microbiota from healthy or unhealthy individuals (normally with dysbiosis), we obtain mice with human-associated microbiota (HMA).

The accuracy of GF technology lies in the ability to control the composition of the environment in which an organism develops and functions (Falk et al., 1998). To this end, gnotobiotic facilities eliminate microbes present in food, water, and bedding through specific sterilization protocols. Typically, these materials are heated to temperatures above 100°C in order to kill bacteria and viruses. Possible contamination is prevented through a fail-safe double-door system, in which autoclaved supply cylinders are docked to a double-door port built into the isolator wall. These security measures enable inoculation by a single microorganism of interest, as well as the consequent reproduction of mono-associated animals, known as monoxenics. Animals inoculated with two microorganisms are known as dixenics, those with three are trixenics, and so on (**Figure 1A**) In the 1960s, Schaedler (Rockefeller University) investigated the effects of different mixtures of bacteria in GF mice. He was the first to study how a specific bacterium colonizes the gut of initially GF mice (Schaedler et al., 1965a,b), and his descriptions of what subsequently became known as the “Schaedler flora” have served as important tools in standardizing the microbiota of laboratory populations of rodents around the world (Schaedler et al., 1965a,b). This microbiota has recently been updated to include extremely oxygen-sensitive (EOS) bacteria, which were not included in the first survey due to the technical difficulties inherent in their isolation (Wymore Brand et al., 2015). Nowadays, this altered Schaedler microbiota (ASF) is used in several laboratories around the world for the study of bacteria-host dynamics (Wymore Brand et al., 2015). In addition, the ability to control the microbiota also enables the transfer of a simplified or complete human microbiota to the GF animals in order to create human-like conditions (Goodman et al., 2011) (**Figure 1B**). However, if the transferred microbiota is not completely defined, the resulting animals are not considered gnotobiotic animals.

Typically, gnotobiotic animals are used to study the effects of different members of the microbiota on the host. As is the case with most biological research (Williams, 2014), the field of gnotobiotics began with studies in which the system of interest—in this case, the microbiota of various animals such as mice, rats, guinea pigs, and chicks—was removed in order to examine its role in different processes (Reyniers, 1959).

Studies with these animals have demonstrated that the crosstalk between microbes and their host is essential for the well-being of the host, and have indicated that the physiology of healthy host individuals is dependent on the composition of the gut microbiota. Indeed, changes in the gut microbiota affect processes as varied as epithelial cell renewal, differentiation, and architecture (Cherbuy et al., 2010); intestinal motility (Falk et al., 1998); and host glycosylation patterns and gene expression (Hooper et al., 2001). The study of gnotobiotic rodents has also allowed researchers to identify and characterize key microbes responsible for the development of the intestinal immune system (Umesaki and Setoyama, 2000; Umesaki, 2014). Thanks to gnotobiology, it is now widely recognized that the microbiome serves its hosts in capacities that go far beyond its role in food digestion. GF animals have dramatic alterations in practically every phenotype that has been studied, including the immune system, brain development, metabolism, behavior, and the function of the heart, lungs, and lymph nodes (Williams, 2014). In addition, the combination of gnotobiotic techniques with other new approaches has revealed causative associations between alterations in the commensal microbiota and diseases such as inflammatory bowel disease (IBD), obesity, and multiple sclerosis, among others (Balish and Warner, 2002; Ley et al., 2006; Turnbaugh et al., 2006; Berer et al., 2011).

## Why Use Gnotobiotic Rodents in Studies of Microbe–Microbe Interactions?

In the mammalian gut, microbes exist in a complex network of cooperation and competition. Simplified experimental culture conditions, in many cases, do not adequately represent the *in vivo* activities and connections among different members of the network, which prevents accurate evaluations of their individual contributions to overall microbiota functionality (Stecher et al., 2013a). Instead, gnotobiology offers a model in which microbe–microbe interactions can be assessed directly in a more complex environment. Furthermore, the use of initially GF animals enables complete knowledge of the microbial composition of a host, with consequent advantages for the interpretation of results compared to studies of conventional mice. For all these reasons, gnotobiotic studies offer a wide range of advantages compared to both other animal models and *in vitro* conditions. Gnotobiotics represents the optimal compromise between the realistic complexity of conventional rodents and the controlled nature of *in vitro* tests, without the incomplete knowledge and control of the environment inherent in the former and the oversimplifications of the latter.

### Gnotobiotics as a Model of Synergistic Microbial Interactions (Cooperative Network Analysis)

The diverse intestinal microbiota is characterized by extensive synergistic ecological interactions (Stecher et al., 2013a). Gnotobiotic animals have been employed for deeper analyses of these important microbial interactions, in particular (i) bacterial succession, (ii) cross-feeding, and (iii) phage regulation of bacterial populations.

Most studies of bacterial succession have focused on the initial gut colonization process (primo-colonization; Fanaro et al., 2003). In these studies, different bacteria are introduced (sequentially or not) into GF animals in order to mimic the progressive colonization of the sterile intestine that begins immediately after birth. For instance, Tomas et al. (2013) compared the transfer of two different microbiota to GF rats: one from suckling rats, rich in early colonizing bacteria, and the other obtained from adult rats, representative of a mature microbiota. In rats with the early colonizing microbiota, strictly anaerobic bacteria succeeded aero-tolerant bacteria. Because of this coordinated process of bacterial succession, at the end of the experiment the microbiotas of both groups had converged on the same species profile, that of the mature inoculum. Similar results have been described for the process of microbiota acquisition in conventional mice (Gillilland et al., 2012). However, the mechanisms behind this succession remain poorly described, as this study focused only on host responses and not the evolution in microbe–microbe interactions (Tomas et al., 2013).

**FIGURE 2:**
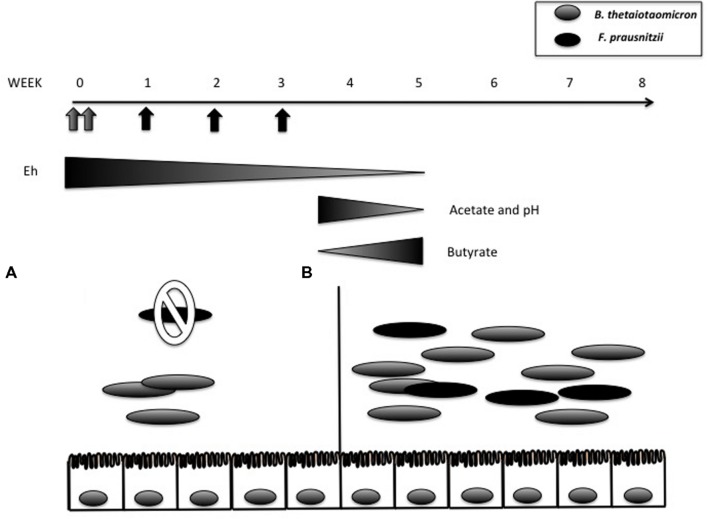
***Bacteroides thetaiotaomicron* (gray oval) and *Faecalibacterium prausnitzii* (black oval) di-associated mice production process. (A)** When *B. thetaiotaomicron* and *F. prausnitzii* were inoculated individually (gray arrows), only *B. thetaiotaomicron* colonized. **(B)** *F. prausnitzii* was successfully implanted after week 4 following three weekly inoculations (black arrows). The oxido-reductive potential (measured by Eh value) in the cecum progressively decreased in the presence of *B. thetaiotaomicron* (from 178 mV in GF rats to 141.1 mV at day 2) and decreased further in the presence of *F. praustnizii* (-274 mV at day 30). Comparison of *B. thetaiotaomicron* mono-associated rats with dixenic rats revealed that in the presence of *F. praustnizii* there was a decrease in pH and acetate and an increase in butyrate. Adapted from Wrzosek et al. (2013).

Aero-tolerant bacteria are thought to “prepare” the colonic environment for the growth of the strictly anaerobic bacteria that will later predominate. Researchers have taken advantage of this phenomenon to introduce certain strictly anaerobic bacteria into mice with a controlled microbiota. For example, the bacterium *Faecalibacterium prausnitzii* has well-known anti-inflammatory properties that contribute to its role as a sensor and promoter of intestinal health (Miquel et al., 2013, 2014). However, because it is EOS, it is quite difficult to obtain monoxenic *F. prausnitzii*-associated rodents. By preparing the environment with *Escherichia coli*, though, Miquel et al. (2015) were able to generate gnotobiotic mice that harbored both *F. prausnitzii* and *E. coli*. Similarly, dixenic *F. prausnitzii*-associated rats were also obtained by first inoculating the rats with *Bacteroides thetaiotaomicron* (Wrzosek et al., 2013) (**Figure 2**). *B. thetaiotaomicron* is an anaerobic bacterium; rather, it decreases the oxido-reductive potential of the gastrointestinal tract (GIT), thus enabling colonization by *F. prausnitzii*. In addition, *B. thetaiotaomicron* and *F. prausnitzii* complement each other metabolically: the former is acetate-producing whereas the latter is acetate-consuming and butyrate-producing (Duncan et al., 2002; Mahowald et al., 2009). In di-associated rats, *B. thetaiotaomicron* produces acetate and *F. prausnitzii* transforms this into butyrate (Wrzosek et al., 2013). These short-chain fatty acids (SCFAs) are also able to stimulate the host response. For instance, SCFA trigger pleiotropic signals in the host, including signals regulating mucin synthesis and secretion (Gaudier et al., 2004; Burger-van Paassen et al., 2009). Both of these dixenic models illustrate the importance of bacterial interactions during the colonization of GF rodents by microorganisms. Since strictly anaerobic bacteria are major components of the intestinal microbiota, these mutualistic relationships are likely of vital importance in microbiota acquisition and maintenance.

As mentioned above, the study of bacterial cross-feeding phenomena is one area in which the tools of gnotobiology will continue to be indispensable. Special models of human microbiota have been developed for this purpose, as only 15% of the bacterial species found in mice are also present in humans (Tomas et al., 2012). Human microbiota-associated (HMA) mice have also been employed as models in the study of the ecology and metabolism of the human intestinal microbiota (Hirayama and Itoh, 2005). For instance, studies of GF mice colonized by human microbiota have clarified the cross-feeding activities of *Akkermansia muciniphila* (Van den Abbeele et al., 2011), a highly specialized bacterium capable of utilizing mucus as a sole carbon and nitrogen source (Derrien et al., 2004; Everard et al., 2013). The mucus-degrading ability of *A. muciniphila* and its localization within the mucus layer have revealed its specific niche and function within the gut (Swidsinski et al., 2009; Png et al., 2010). By degrading mucus, *A. muciniphila* has the ability to produce SCFAs (acetate and propionate) (Derrien et al., 2004), which can stimulate microbiota interactions and a host response. Specifically, oligosaccharides and acetate stimulate growth and metabolic activity in commensal bacteria growing close to the mucus layer, which may in turn discourage pathogenic bacteria from crossing the mucus layer to reach the intestinal cells (Derrien et al., 2004, 2010, 2011). Furthermore, an increase in acetate and propionate due to the degradation of mucus by *A. muciniphila* could stimulate other bacterial groups with well-known butyrate-producing capacities as it has been proved *in vitro* with other acetate producers (Rios-Covian et al., 2015).

Other scientific research on the human microbiome has stimulated interest in probiotics, health-promoting agents that are able to modulate the intestinal microbiota. In a recent experiment, HFA mice were used to study the ability of *Bifidobacterium longum* strain BB536 to modulate the gut environment (Sugahara et al., 2015). HFA mice that were supplemented with strain BB536 showed increased fecal levels of butyrate and pimelate, a precursor of biotin. In addition, the BB536-supplemented mice also had metabolic alterations related to biotin synthesis, specifically involving *Bacteroides caccae* and the increased prevalence of *Eubacterium rectale*, a butyrate producer (Sugahara et al., 2015). All these possible interactions of *B. longum* BB536 with the microbial community might explain, at least in part, various reports of its beneficial physiological effects on the host, including anti-allergy effects, competition against harmful bacteria, and improvement in defecation frequency and stool characteristics (Odamaki et al., 2007, 2012; Xiao et al., 2007; Namba et al., 2010).

Gnotobiotics also offers clear advantages for the study of phage-bacteria dynamics in the human gut. Bacterial viruses (phages) are the most abundant biological group on earth (Rodriguez-Valera et al., 2009). In some ecosystems, they are capable of maintaining high levels of diversity of bacterial strains through lysis of their hosts, in a process described by the constant-diversity dynamics model of bacterial diversity; this model is based on the fact that many of the genes that differ between strains affect regions that are potential phage recognition targets (Rodriguez-Valera et al., 2009). Recently, Reyes and coworkers introduced sequenced human gut bacteria into GF mice and followed this with a controlled phage attack using virus-like particles (VLPs) purified from the fecal microbiota (Rodriguez-Valera et al., 2009). The authors observed transient changes in bacterial community structure and bacterial acquisition of resistance to phage attack through both ecological and epigenetic mechanisms. These results illustrate the utility of gnotobiotic mice in characterizing ecological relationships among all components in the gut, viruses as well as bacteria.

### Gnotobiotic Animals as Models of Antagonistic Microbial Interactions (Competitive Network Analysis)

Microbe–microbe interactions are not always cooperative. Antagonistic cross-talk is a fundamental link in microbial ecology and vital for the maintenance of host health. Gnotobiotic animals have been employed to obtain deeper insight into the ability of the microbiota to protect against infections, a process termed colonization resistance (Stecher et al., 2013b).

Alterations in microbiota homeostasis generally result in dysbiosis, an imbalance among bacterial species that often occurs in the digestive tract. This condition, also known as dysbacteriosis, is associated with illnesses such as IBD, chronic obesity, cancer, and bacterial vaginosis (Martin et al., 2013, 2014a,b). Efforts to link these diseases to specific changes in the microbiota have been hindered, though, by a lack of knowledge of healthy bacterial communities. Indeed, it is not well understood if dysbiosis itself is a consequence or a cause of an imbalance in bacteria–bacteria relationships in the gastrointestinal tract.

When colonization resistance is disrupted through antibiotic-mediated perturbations in the microbiota or other similar phenomena, infections by nosocomial pathogens tend to increase (Ubeda and Pamer, 2012; Stecher et al., 2013b). This observation could be explained by the 1983 proposal of Freter et al. (1983a,b) that populations of most intestinal bacteria are held in check by competition for limited substrates. GF animals provide an ideal testing ground in which to explore this hypothesis, as the level of competition within a particular host can be manipulated through the introduction of varying numbers of bacterial strains. Studies using this approach have found that, because they lack competing bacterial species, GF animals are more susceptible than conventional ones to infections by *E. coli, Clostridium difficile, Vibrio cholerae*, or *Citrobacter rodentium* (Collins and Carter, 1978; Butterton et al., 1996; Stecher et al., 2005; Kamada et al., 2012; Reeves et al., 2012). This hypothesis was further supported by experiments in which the transplantation of healthy fecal microbiota increased host resistance (Barman et al., 2008; Endt et al., 2010; Lawley et al., 2012). Thus, Freter’s theory provides a straightforward explanation of why gnotobiotic mice are more sensitive to nosocomial infections: the lack of substrate competition reduces barriers to colonization. However, this conclusion is probably over-simplistic. Nowadays, studies from various fields, including gnotobiology, have concluded that colonization resistance is the result of not only a highly complex interplay among members of the commensal microbiota, but also of interactions among the microbiome [the entire community of microbes and their genetic material; (Martin et al., 2014a), the intestinal mucosa, and the immune system (Stecher et al., 2013b)]. For example, GF mice have been used to examine how individual bacteria species or bacterial communities influence colonization resistance to *C. difficile*. In particular, GF mice that had been inoculated only with a murine isolate from the family *Lachnospiraceae* demonstrated partially restored colonization resistance to *C. difficile* (Reeves et al., 2012), confirming that the indigenous microbial community of the GIT and the inter-relationships among its members determine susceptibility to colonization and growth of *C. difficile* (Reeves et al., 2011, 2012). These results highlight the potential of gnotobiotic mice as a controlled environment in which to study antagonist microbial interactions.

Colonization resistance is not the only example of antagonistic interactions in the microbiota. For example, gnotobiotic models have also been used to analyze growth competition between the well-known yoghurt bacteria *Streptococcus thermophilus* and *Lactobacillus delbrueckii* ssp. *bulgaricus*. The growth and lactate production of these two bacteria were monitored in different media and in the GIT of GF rats. When GF rats were not supplemented with lactose, they were colonized only by *S. thermophilus*, not by *L. bulgaricus*. However, when the rats’ drinking water was supplemented with lactose, both bacteria were able to colonize. Interestingly, when lactose-supplemented GF rats were inoculated with a mix of both bacteria, *S. thermophilus* colonized the GIT faster and more extensively than *L. bulgaricus* did. This work showed that *S. thermophilus* has a competitive and growth advantage over *L. bulgaricus in vitro* as well as *in vivo* in the GIT of GF rats (Ben-Yahia et al., 2012).

## Future Trends in Gnotobiology

At this point, gnotobiology is a mature science with applications that go far beyond conventional ones. The classical experiments and protocols used routinely in the study of microbes’ interactions with their hosts can easily be modified in order to investigate interactions between and among multiple members of the microbiota. As an example, a study aiming to understand the effect of a microbe of interest on a particular disease would benefit greatly from the use of mice with a controlled, described microbiota. Such mice would then also be the perfect experimental system in which to analyze the relationship of the same microbe with the rest of the microbiota. Furthermore, this analysis could be performed in both healthy and diseased hosts. Similarly, our understanding of a pathogen’s activity in a host could be enhanced through the use of various monoxenic mice: such mice could be infected with a pathogen and monitored in order to directly assess the effect of individual resident microorganisms on the disease caused by the pathogen. This model presents unique opportunities to analyze the possible antagonistic interactions between resident and invading microorganisms.

In brief, the options with gnotobiology are almost unlimited. Furthermore, the combination of gnotobiotics with new-*omics* approaches (e.g., transcriptomics, metabolomics, and genomics) and molecular genetics (genetically modified mice and bacteria) could provide important insights into the roles of microbe–microbe interactions. This knowledge could improve our understanding of how commensal bacteria change into opportunistic pathogens, how pathogens are able to proliferate, and how probiotic bacteria could help to resolve dysbiosis.

## Concluding Remarks

Here, we have summarized the most important outcomes of gnotobiological research in the field of microbe–microbe interactions as well as other concerns with relevance to the field. The most significant questions in microbiota analysis, including microbial interaction, rely on gnotobiotic animals for further advancements in the field. GF animals will be indispensable in future investigations of how certain microorganisms are able to colonize and survive in the host, while others, e.g., pathogens, are not. Studies of interaction and communication within the microbiota will also depend heavily on gnotobiotic approaches.

## Author Contributions

RM, LB-H, and PL designed the mini-review. RM wrote the manuscript. LB-H and PL corrected the manuscript. All the authors approved the final version of the manuscript.

## Conflict of Interest Statement

The authors declare that the research was conducted in the absence of any commercial or financial relationships that could be construed as a potential conflict of interest.

## References

[B1] Balish E. Warner T. (2002). Enterococcus faecalis induces inflammatory bowel disease in interleukin-10 knockout mice. *Am. J. Pathol.* 160 2253–2257. 10.1016/S0002-9440(10)61172-8 12057927 PMC1850822

[B2] Barman M. Unold D. Shifley K. Amir E. Hung K. Bos N. (2008). Enteric salmonellosis disrupts the microbial ecology of the murine gastrointestinal tract. *Infect. Immun.* 76 907–915. 10.1128/IAI.01432-07 18160481 PMC2258829

[B3] Ben-Yahia L. Mayeur C. Rul F. Thomas M. (2012). Growth advantage of *Streptococcus thermophilus* over *Lactobacillus bulgaricus* in vitro and in the gastrointestinal tract of gnotobiotic rats. *Benef. Microbes* 3 211–219. 10.3920/BM2012.0012 22968410

[B4] Berer K. Mues M. Koutrolos M. Rasbi Z. A. Boziki M. Johner C. (2011). Commensal microbiota and myelin autoantigen cooperate to trigger autoimmune demyelination. *Nature* 479 538–541. 10.1038/nature10554 22031325

[B5] Burger-van Paassen N. Vincent A. Puiman P. J. van der Sluis M. Bouma J. Boehm G. (2009). The regulation of intestinal mucin MUC2 expression by short-chain fatty acids: implications for epithelial protection. *Biochem. J.* 420 211–219. 10.1042/BJ20082222 19228118

[B6] Butterton J. R. Ryan E. T. Shahin R. A. Calderwood S. B. (1996). Development of a germfree mouse model of *Vibrio cholerae* infection. *Infect. Immun.* 64 4373–4377. 8926115 10.1128/iai.64.10.4373-4377.1996 PMC174383

[B7] Cherbuy C. Honvo-Houeto E. Bruneau A. Bridonneau C. Mayeur C. Duee P. H. (2010). Microbiota matures colonic epithelium through a coordinated induction of cell cycle-related proteins in gnotobiotic rat. *Am. J. Physiol. Gastrointest. Liver Physiol.* 299 G348–G357. 10.1152/ajpgi.00384.2009 20466941

[B8] Collins F. M. Carter P. B. (1978). Growth of *Salmonella*e in orally infected germfree mice. *Infect. Immun.* 21 41–47. 361568 10.1128/iai.21.1.41-47.1978 PMC421954

[B9] Derrien M. Van Baarlen P. Hooiveld G. Norin E. Muller M. de Vos W. M. (2011). Modulation of mucosal immune response, tolerance, and proliferation in mice colonized by the mucin-degrader *Akkermansia muciniphila*. *Front. Microbiol.* 2:166 10.3389/fmicb.2011.00166 PMC3153965 21904534

[B10] Derrien M. van Passel M. W. van de Bovenkamp J. H. Schipper R. G. de Vos W. M. Dekker J. (2010). Mucin-bacterial interactions in the human oral cavity and digestive tract. *Gut Microbes* 1 254–268. 10.4161/gmic.1.4.12778 21327032 PMC3023607

[B11] Derrien M. Vaughan E. E. Plugge C. M. de Vos W. M. (2004). *Akkermansia muciniphila* gen. nov., sp. nov., a human intestinal mucin-degrading bacterium. *Int. J. Syst. Evol. Microbiol.* 54 1469–1476. 10.1099/ijs.0.02873-054/5/1469 15388697

[B12] Duncan S. H. Hold G. L. Harmsen H. J. Stewart C. S. Flint H. J. (2002). Growth requirements and fermentation products of *Fusobacterium prausnitzii*, and a proposal to reclassify it as *Faecalibacterium prausnitzii* gen. nov., comb. nov. *Int. J. Syst. Evol. Microbiol.* 52 2141–2146. 10.1099/00207713-52-6-2141 12508881

[B13] Endt K. Stecher B. Chaffron S. Slack E. Tchitchek N. Benecke A. (2010). The microbiota mediates pathogen clearance from the gut lumen after non-typhoidal *Salmonella* diarrhea. *PLoS Pathog.* 6:e1001097 10.1371/journal.ppat.1001097 PMC2936549 20844578

[B14] Everard A. Belzer C. Geurts L. Ouwerkerk J. P. Druart C. Bindels L. B. (2013). Cross-talk between *Akkermansia muciniphila* and intestinal epithelium controls diet-induced obesity. *Proc. Natl. Acad. Sci. U.S.A.* 110 9066–9071. 10.1073/pnas.1219451110 23671105 PMC3670398

[B15] Falk P. G. Hooper L. V. Midtvedt T. Gordon J. I. (1998). Creating and maintaining the gastrointestinal ecosystem: what we know and need to know from gnotobiology. *Microbiol. Mol. Biol. Rev.* 62 1157–1170. 9841668 10.1128/mmbr.62.4.1157-1170.1998 PMC98942

[B16] Fanaro S. Chierici R. Guerrini P. Vigi V. (2003). Intestinal microflora in early infancy: composition and development. *Acta Paediatr. Suppl.* 91 48–55. 14599042 10.1111/j.1651-2227.2003.tb00646.x

[B17] Freter R. Brickner H. Botney M. Cleven D. Aranki A. (1983a). Mechanisms that control bacterial populations in continuous-flow culture models of mouse large intestinal flora. *Infect. Immun.* 39 676–685. 6339388 10.1128/iai.39.2.676-685.1983 PMC348004

[B18] Freter R. Brickner H. Fekete J. Vickerman M. M. Carey K. E. (1983b). Survival and implantation of *Escherichia coli* in the intestinal tract. *Infect. Immun.* 39 686–703. 6339389 10.1128/iai.39.2.686-703.1983 PMC348005

[B19] Gaudier E. Jarry A. Blottiere H. M. de Coppet P. Buisine M. P. Aubert J. P. (2004). Butyrate specifically modulates MUC gene expression in intestinal epithelial goblet cells deprived of glucose. *Am. J. Physiol. Gastrointest. Liver Physiol.* 287 G1168–G1174. 10.1152/ajpgi.00219.2004 15308471

[B20] Gillilland M. G. I. I. I. Erb-Downward J. R. Bassis C. M. Shen M. C. Toews G. B. Young V. B. (2012). Ecological succession of bacterial communities during conventionalization of germ-free mice. *Appl. Environ. Microbiol.* 78 2359–2366. 10.1128/AEM.05239-11 22286988 PMC3302583

[B21] Goodman A. L. Kallstrom G. Faith J. J. Reyes A. Moore A. Dantas G. (2011). Extensive personal human gut microbiota culture collections characterized and manipulated in gnotobiotic mice. *Proc. Natl. Acad. Sci. U.S.A.* 108 6252–6257. 10.1073/pnas.1102938108 21436049 PMC3076821

[B22] Gustafsson B. E. (1959). Lightweight stainless steel systems for rearing germfree animals. *Ann. N. Y. Acad. Sci.* 78 17–28. 10.1111/j.1749-6632.1959.tb53092.x 13830425

[B23] Hirayama K. Itoh K. (2005). Human flora-associated (HFA) animals as a model for studying the role of intestinal flora in human health and disease. *Curr. Issues Intest. Microbiol.* 6 69–75. 16107039

[B24] Hooper L. V. Wong M. H. Thelin A. Hansson L. Falk P. G. Gordon J. I. (2001). Molecular analysis of commensal host-microbial relationships in the intestine. *Science* 291 881–884. 10.1126/science.291.5505.881 11157169

[B25] Kamada N. Kim Y. G. Sham H. P. Vallance B. A. Puente J. L. Martens E. C. (2012). Regulated virulence controls the ability of a pathogen to compete with the gut microbiota. *Science* 336 1325–1329. 10.1126/science.1222195 22582016 PMC3439148

[B26] Lawley T. D. Clare S. Walker A. W. Stares M. D. Connor T. R. Raisen C. (2012). Targeted restoration of the intestinal microbiota with a simple, defined bacteriotherapy resolves relapsing *Clostridium difficile* disease in mice. *PLoS Pathog.* 8:e1002995 10.1371/journal.ppat.1002995 PMC3486913 23133377

[B27] Ley R. E. Turnbaugh P. J. Klein S. Gordon J. I. (2006). Microbial ecology: human gut microbes associated with obesity. *Nature* 444 1022–1023. 10.1038/4441022a 17183309

[B28] Mahowald M. A. Rey F. E. Seedorf H. Turnbaugh P. J. Fulton R. S. Wollam A. (2009). Characterizing a model human gut microbiota composed of members of its two dominant bacterial phyla. *Proc. Natl. Acad. Sci. U.S.A.* 106 5859–5864. 10.1073/pnas.0901529106 19321416 PMC2660063

[B29] Martin R. Miquel S. Langella P. Bermudez-Humaran L. G. (2014a). The role of metagenomics in understanding the human microbiome in health and disease. *Virulence* 5 413–423. 10.4161/viru.27864 24429972 PMC3979869

[B30] Martin R. Miquel S. Ulmer J. Kechaou N. Langella P. Bermudez-Humaran L. G. (2013). Role of commensal and probiotic bacteria in human health: a focus on inflammatory bowel disease. *Microb. Cell Fact.* 12 71 10.1186/1475-2859-12-71 PMC3726476 23876056

[B31] Martin R. Miquel S. Ulmer J. Langella P. Bermudez-Humaran L. G. (2014b). Gut ecosystem: how microbes help us. *Benef. Microbes* 5 219–233. 10.3920/BM2013.0057 24583612

[B32] Miquel S. Leclerc M. Martin R. Chain F. Lenoir M. Raguideau S. (2015). Identification of metabolic signatures linked to anti-inflammatory effects of *Faecalibacterium prausnitzii*. *Mbio* 6 e00300–e00315. 10.1128/mBio.00300-15 25900655 PMC4453580

[B33] Miquel S. Martin R. Bridonneau C. Robert V. Sokol H. Bermudez-Humaran L. G. (2014). Ecology and metabolism of the beneficial intestinal commensal bacterium *Faecalibacterium prausnitzii*. *Gut Microbes* 5 146–151. 10.4161/gmic.27651 24637606 PMC4063839

[B34] Miquel S. Martin R. Rossi O. Bermudez-Humaran L. G. Chatel J. M. Sokol H. (2013). *Faecalibacterium prausnitzii* and human intestinal health. *Curr. Opin. Microbiol.* 16 255–261. 10.1016/j.mib.2013.06.003 23831042

[B35] Namba K. Hatano M. Yaeshima T. Takase M. Suzuki K. (2010). Effects of *Bifidobacterium longum* BB536 administration on influenza infection, influenza vaccine antibody titer, and cell-mediated immunity in the elderly. *Biosci. Biotechnol. Biochem.* 74 939–945. 10.1271/bbb.90749 20460726

[B36] Odamaki T. Sugahara H. Yonezawa S. Yaeshima T. Iwatsuki K. Tanabe S. (2012). Effect of the oral intake of yogurt containing *Bifidobacterium longum* BB536 on the cell numbers of enterotoxigenic *Bacteroides fragilis* in microbiota. *Anaerobe* 18 14–18. 10.1016/j.anaerobe.2011.11.004 22138361

[B37] Odamaki T. Xiao J. Z. Iwabuchi N. Sakamoto M. Takahashi N. Kondo S. (2007). Influence of *Bifidobacterium longum* BB536 intake on faecal microbiota in individuals with Japanese cedar pollinosis during the pollen season. *J. Med. Microbiol.* 56 1301–1308. 10.1099/jmm.0.47306-0 17893165

[B38] Png C. W. Linden S. K. Gilshenan K. S. Zoetendal E. G. McSweeney C. S. Sly L. I. (2010). Mucolytic bacteria with increased prevalence in IBD mucosa augment in vitro utilization of mucin by other bacteria. *Am. J. Gastroenterol.* 105 2420–2428. 10.1038/ajg.2010.281 20648002

[B39] Reeves A. E. Koenigsknecht M. J. Bergin I. L. Young V. B. (2012). Suppression of *Clostridium difficile* in the gastrointestinal tracts of germfree mice inoculated with a murine isolate from the family Lachnospiraceae. *Infect. Immun.* 80 3786–3794. 10.1128/IAI.00647-12 22890996 PMC3486043

[B40] Reeves A. E. Theriot C. M. Bergin I. L. Huffnagle G. B. Schloss P. D. Young V. B. (2011). The interplay between microbiome dynamics and pathogen dynamics in a murine model of *Clostridium difficile* Infection. *Gut Microbes* 2 145–158. 10.4161/gmic.2.3.16333 21804357 PMC3225775

[B41] Reyniers J. A. (1959). The pure culture concept and gnotobiotics. *Ann. N. Y. Acad. Sci.* 78 3–16.

[B42] Rios-Covian D. Gueimonde M. Duncan S. H. Flint H. J. de Los Reyes-Gavilan C. G. (2015). Enhanced butyrate formation by cross-feeding between *Faecalibacterium prausnitzii* and *Bifidobacterium adolescentis*. *FEMS Microbiol. Lett.* 362:fnv176 10.1093/femsle/fnv176 26420851

[B43] Rodriguez-Valera F. Martin-Cuadrado A. B. Rodriguez-Brito B. Pasic L. Thingstad T. F. Rohwer F. (2009). Explaining microbial population genomics through phage predation. *Nat. Rev. Microbiol.* 7 828–836. 10.1038/nrmicro2235 19834481

[B44] Schaedler R. W. Dubos R. Costello R. (1965a). Association of germfree mice with bacteria isolated from normal mice. *J. Exp. Med.* 122 77–82. 10.1084/jem.122.1.77 14325475 PMC2138033

[B45] Schaedler R. W. Dubos R. Costello R. (1965b). The development of the bacterial flora in the gastrointestinal tract of mice. *J. Exp. Med.* 122 59–66. 10.1084/jem.122.1.59 14325473 PMC2138024

[B46] Stecher B. Berry D. Loy A. (2013a). Colonization resistance and microbial ecophysiology: using gnotobiotic mouse models and single-cell technology to explore the intestinal jungle. *FEMS Microbiol. Rev.* 37 793–829. 10.1111/1574-6976.12024 23662775

[B47] Stecher B. Macpherson A. J. Hapfelmeier S. Kremer M. Stallmach T. Hardt W. D. (2005). Comparison of *Salmonella enterica* serovar Typhimurium colitis in germfree mice and mice pretreated with streptomycin. *Infect. Immun.* 73 3228–3241. 10.1128/IAI.73.6.3228-3241.2005 15908347 PMC1111827

[B48] Stecher B. Maier L. Hardt W. D. (2013b). ‘Blooming’ in the gut: how dysbiosis might contribute to pathogen evolution. *Nat. Rev. Microbiol.* 11 277–284. 10.1038/nrmicro2989 23474681

[B49] Sugahara H. Odamaki T. Fukuda S. Kato T. Xiao J. Z. Abe F. (2015). *Bifidobacterium longum* alters gut luminal metabolism through modification of the gut microbial community. *Sci. Rep.* 5:13548 10.1038/srep13548 PMC4552000 26315217

[B50] Swidsinski A. Ung V. Sydora B. C. Loening-Baucke V. Doerffel Y. Verstraelen H. (2009). Bacterial overgrowth and inflammation of small intestine after carboxymethylcellulose ingestion in genetically susceptible mice. *Inflamm. Bowel Dis.* 15 359–364. 10.1002/ibd.20763 18844217

[B51] Tomas J. Langella P. Cherbuy C. (2012). The intestinal microbiota in the rat model: major breakthroughs from new technologies. *Anim. Health Res. Rev.* 13 54–63. 10.1017/S1466252312000072 22853927

[B52] Tomas J. Wrzosek L. Bouznad N. Bouet S. Mayeur C. Noordine M. L. (2013). Primocolonization is associated with colonic epithelial maturation during conventionalization. *FASEB J.* 27 645–655. 10.1096/fj.12-216861 23118025

[B53] Turnbaugh P. J. Ley R. E. Mahowald M. A. Magrini V. Mardis E. R. Gordon J. I. (2006). An obesity-associated gut microbiome with increased capacity for energy harvest. *Nature* 444 1027–1031. 10.1038/nature05414 17183312

[B54] Ubeda C. Pamer E. G. (2012). Antibiotics, microbiota, and immune defense. *Trends Immunol.* 33 459–466. 10.1016/j.it.2012.05.003 22677185 PMC3427468

[B55] Umesaki Y. (2014). Use of gnotobiotic mice to identify, and characterize key microbes responsible for the development of the intestinal immune system. *Proc. Jpn. Acad. Ser. B Phys. Biol. Sci.* 90 313–332. 10.2183/pjab.90.313 PMC4324924 25391317

[B56] Umesaki Y. Setoyama H. (2000). Structure of the intestinal flora responsible for development of the gut immune system in a rodent model. *Microbes Infect.* 2 1343–1351. 10.1016/S1286-4579(00)01288-0 11018451

[B57] Van den Abbeele P. Gerard P. Rabot S. Bruneau A. El Aidy S. Derrien M. (2011). Arabinoxylans and inulin differentially modulate the mucosal and luminal gut microbiota and mucin-degradation in humanized rats. *Environ. Microbiol.* 13 2667–2680. 10.1111/j.1462-2920.2011.02533.x 21883787

[B58] Williams S. C. (2014). Gnotobiotics. *Proc. Natl. Acad. Sci. U.S.A.* 111 1661 10.1073/pnas.1324049111 PMC3918800 24497491

[B59] Wrzosek L. Miquel S. Noordine M. L. Bouet S. Joncquel Chevalier-Curt M. Robert V. (2013). *Bacteroides* thetaiotaomicron and *Faecalibacterium prausnitzii* influence the production of mucus glycans and the development of goblet cells in the colonic epithelium of a gnotobiotic model rodent. *BMC Biol.* 11:61 10.1186/1741-7007-11-61 PMC3673873 23692866

[B60] Wymore Brand M. Wannemuehler M. J. Phillips G. J. Proctor A. Overstreet A. M. Jergens A. E. (2015). The Altered Schaedler Flora: continued applications of a defined murine microbial community. *ILAR J.* 56 169–178. 10.1093/ilar/ilv012 26323627 PMC4554250

[B61] Xiao J. Z. Kondo S. Yanagisawa N. Miyaji K. Enomoto K. Sakoda T. (2007). Clinical efficacy of probiotic *Bifidobacterium longum* for the treatment of symptoms of Japanese cedar pollen allergy in subjects evaluated in an environmental exposure unit. *Allergol. Int.* 56 67–75. 10.2332/allergolint.O-06-455 17259812

